# Supplementary material for: Hypoxia Engineered Bone Marrow Mesenchymal Stem Cells Targeting System with Tumor Microenvironment Regulation for Enhanced Chemotherapy of Breast Cancer
Source: Biomedicines. 2021 May 19;9(5):575. doi: 10.3390/biomedicines9050575 (PMC8160638; doi:10.3390/biomedicines9050575)
Supplement: Supplementary file 1 [file biomedicines-09-00575-s001.zip › biomedicines-1183392-supplementary.pdf]

biomedicines

*Article*

# Hypoxia Engineered Bone Marrow Mesenchymal Stem Cells Targeting System with Tumor Microenvironment Regulation for Enhanced Chemotherapy of Breast Cancer

**Jingzhi Zu [1,†], Liwei Tan [1,2,3,†], Li Yang [4], Qi Wang [2], Jing Qin [2], Jing Peng [2], Hezhong Jiang [2], Rui Tan [1,2,*] and Jian Gu [5,*]**

1 College of Medicine, Southwest Jiaotong University, Chengdu 610031, China; zjz578060490@163.com (J.Z.); rafael0927@163.com (L.T.)
2 College of Life Science and Engineering, Southwest Jiaotong University, Chengdu 610031, China; wangqi20210122@163.com (Q.W.); QJ1473829841@163.com (J.Q.); pj554820@163.com (J.P.); jianghz10@sina.com (H.J.)
3 Sichuan Purity Pharmaceutical Technology Co., Ltd., 3-1 Jiuxing Ave, Hi-Tech Zone, Chengdu 610041, China
4 School of Materials Science and Engineering, Southwest Jiaotong University, Chengdu 610031, China; l.yang2012@foxmail.com
5 School of Pharmacy, Southwest University for Nationalities, Chengdu 610051, China
* Correspondence: tanruiswjtu@sohu.com or tanrui@swjtu.edu.cn (R.T.); gujiancd@163.com (J.G.)
† Jingzhi Zu and Liwei Tan are the co-first authors for this work.

**Table S1.** Drug loading of the complexes with different DTX concentration.

| Concentration (μM) | Intracellular DTX (pg/cell) | |
|---|---|---|
| | DTX@bMSCs | DTX@H-bMSCs |
| 10 | 11.11 ± 0.35 | 10.01 ± 2.15 |
| 20 | 23.59 ± 3.57 | 23.13 ± 4.99 |
| 30 | 73.87 ± 3.33 | 67.87 ± 7.37 |
| 40 | 152.90 ± 0.47 | 145.91 ± 5.54 |
| 50 | 156.32 ± 2.03 | 152.27 ± 7.23 |
| 60 | 172.75 ± 7.93 | 175.15 ± 8.42 |

These samples in each group and the values were mean ± SEM.

**Table S2.** Drug loading of the complexes with 40μM DTX concentration at different times.

| Time (h) | Intracellular DTX (pg/cell) | | | |
|---|---|---|---|---|
| | Free C6@bMSCs | DTX@bMSCs | Free C6@H-bMSCs | DTX@H-bMSCs |
| 0.5 | 3.05 ± 1.43 | 9.74 ± 1.93 | 5.50 ± 1.85 | 13.07 ± 2.30 |
| 1 | 11.53 ± 4.68 | 38.50 ± 7.19 | 13.95 ± 2.51 | 38.61 ± 4.51 |
| 2 | 34.66 ± 4.33 | 72.32 ± 5.46 | 24.90 ± 5.87 | 63.69 ± 3.09 |
| 4 | 57.63 ±12.84 | 135.54 ± 7.23 | 64.02 ± 7.39 | 141.05 ± 10.07 |
| 6 | 66.15 ±8.75 | 148.31 ± 8.22 | 71.05 ± 5.53 | 150.90 ± 8.23 |
| 8 | 76.63 ± 9.06 | 158.34 ± 7.34 | 81.22 ± 7.48 | 158.02 ± 10.55 |

These samples in each group and the values were mean ± SEM.

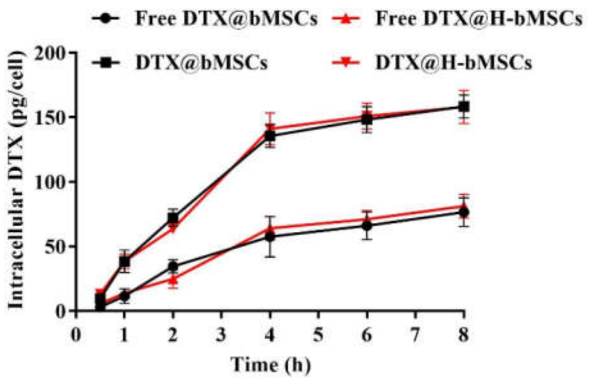


**Figure S1.** Drug loading of the complexes at different times.

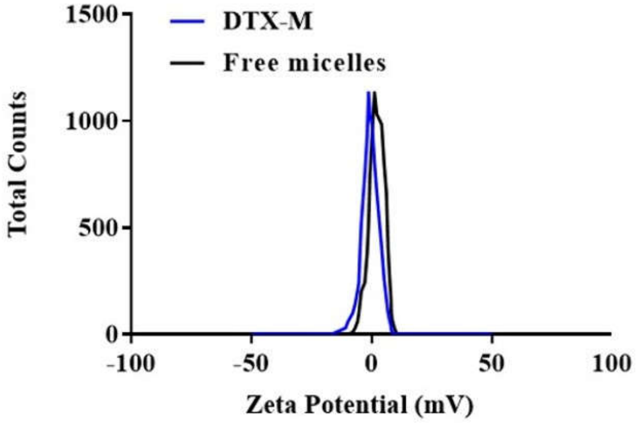


**Figure S2.** The zeta potential of DTX-M and blank micelles.

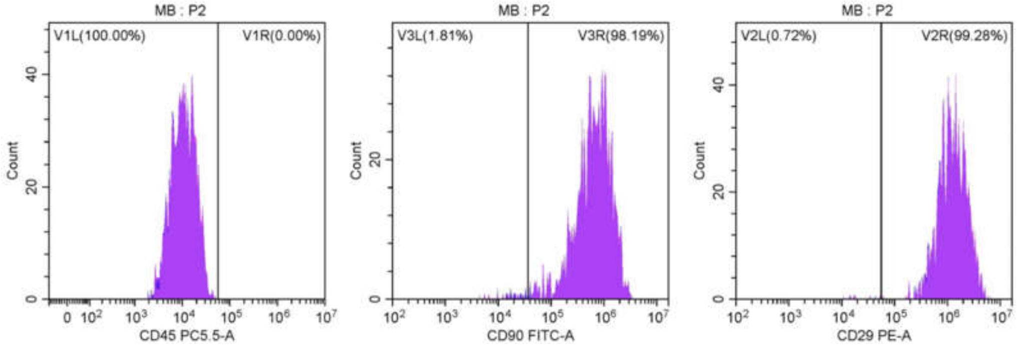


**Figure S3.** Identification of the bMSCs isolated from BALB/c mice by flow cytometry.

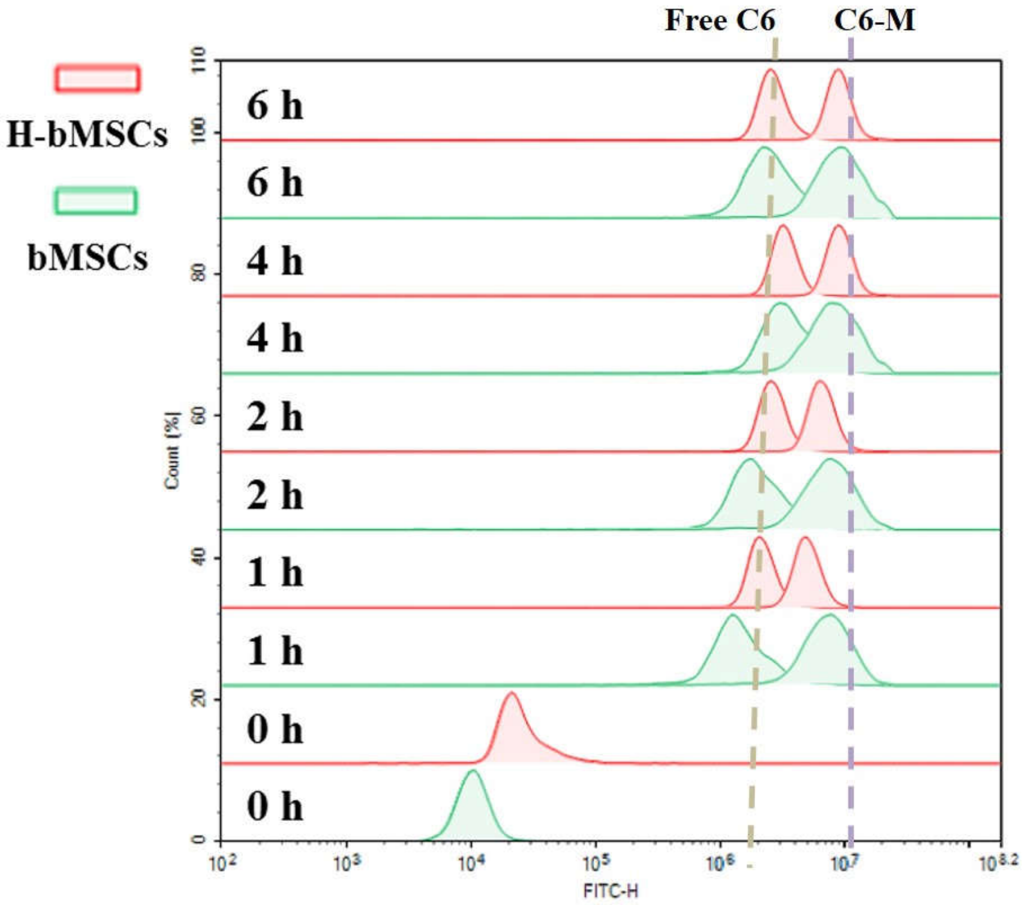


**Figure S4.** Flow cytometry analysis of cellular uptake ability at different co-incubation time.

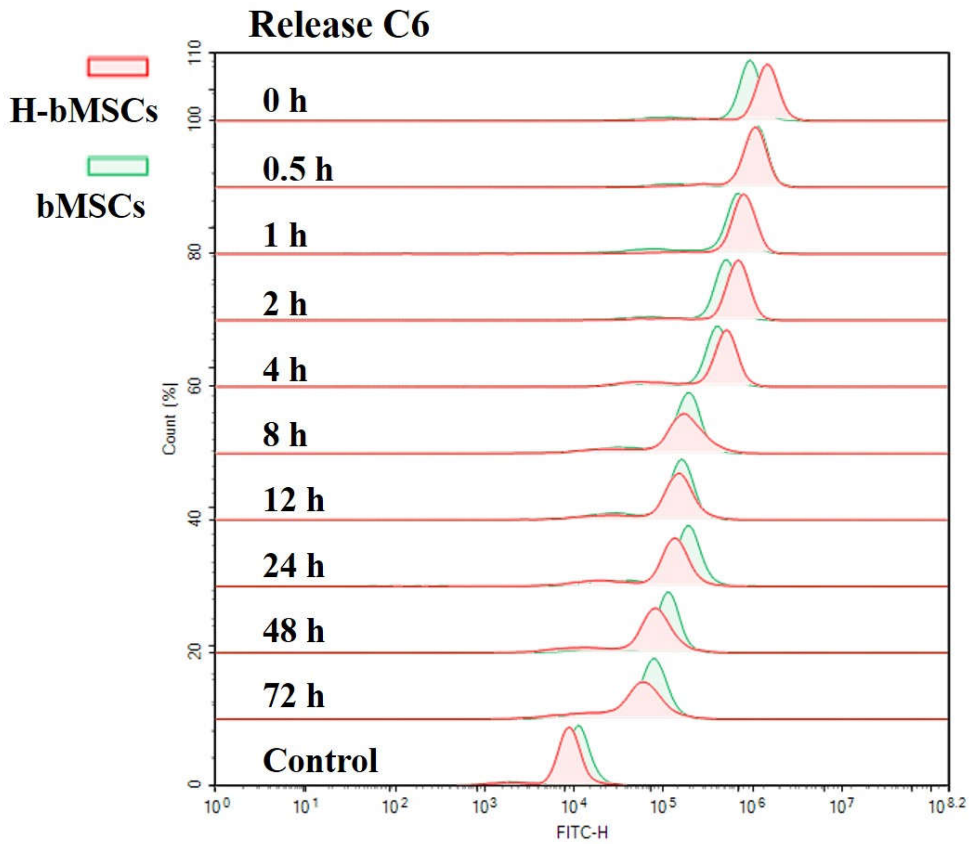


**Figure S5.** Flow cytometry analysis of the release behavior from H-bMSCs or bMSCs.
